# Developing a program theory for implementing a Danish social prescribing model: an ethnographic study

**DOI:** 10.3389/frhs.2026.1815178

**Published:** 2026-08-11

**Authors:** Jeanette Wassar Kirk, Lisa Suvarna Oldrup, Ove Andersen, Per Nilsen, Marie Broholm-Holst

**Affiliations:** 1 Department of Clinical Research, Copenhagen University Hospital, Amager and Hvidovre, Hvidovre, Denmark; 2 Research Group Social Health, National Institute of Public Health, University of Southern Denmark, Copenhagen, Denmark; 3 Emergency Department, Copenhagen University Hospital Hvidovre, Hvidovre, Denmark; 4 Faculty of Health and Medical Sciences, Department of Clinical Medicine, University of Copenhagen, Copenhagen, Denmark; 5 Department of Health, Medicine and Caring Sciences, Linköping University, Linköping, Sweden; 6 School of Health and Welfare, Halmstad University, Halmstad, Sweden

**Keywords:** ethnography, general practitioner, implementation strategies, program theory, social prescribing

## Abstract

**Introduction:**

Social prescribing (SP) is increasingly promoted internationally as a cross-sector strategy to address complex health and social needs by linking primary care patients with community-based, non-clinical support. Despite increasing policy uptake and expanding evaluations of outcomes, evidence on how SP models are implemented and become embedded in routine practice across diverse welfare and organizational contexts remains limited and fragmented. Existing studies tend to catalogue implementation activities and contextual barriers without sufficiently explaining how implementation strategies operate through actors, activate underlying mechanisms, and interact with contextual conditions. We aimed to address this gap by developing an empirically grounded, mechanism-based program theory for implementing a Danish SP model. Specifically, we examined which implementation strategies were distributed across key actor groups, and how strategies performed by specific actors were assumed to activate mechanisms under specific contextual conditions to generate implementation outcomes.

**Methods:**

We conducted an ethnographic field study of the early implementation phase (from mid-2024 to mid-2025) of a Danish SP model (Southwest CPH) initiated by general practitioners (GPs) in Copenhagen in collaboration with link workers (LWs), a Volunteer Centre, and the DaneAge Association. Data comprised 68 days of participant observations supplemented by documents (e-mails, training materials, and reports). Analysis followed an abductive five-step approach: (1) mapping implementation strategies using the ERIC taxonomy, (2) analysing the distribution of strategies across actors, (3) examining systemic interaction patterns across clinical, community, and governance settings, (4) identifying actor logics and mechanisms informed by cultural models, and (5) synthesizing findings into a mechanism-based program theory drawing on realist evaluation, systems theory, and Proctor's implementation outcomes. Findings were iteratively validated through two rounds of analytic dialogue with all project members.

**Results:**

Implementation strategies spanned six ERIC clusters and were distributed across four actor groups in a differentiated yet complementary pattern. GPs primarily led dissemination, policy-related activities, and infrastructural anchoring; LWs concentrated on training, relationship-building, and iterative learning; the Volunteer Centre facilitated local networking and community infrastructure; and the DaneAge Association contributed to national-level advocacy and legitimacy work. Synthesis identified five cross-cutting mechanism families through which strategies were assumed to contribute to implementation outcomes under specific contextual conditions: (1) legitimacy, (2) standardization and normalization, (3) relational sense-making and trust-building, (4) network weaving and coalition formation, and (5) knowledge visibility and mobilization. The resulting program theory specifies how combinations of strategies enacted across actors and system levels are expected to generate key implementation outcomes, including adoption, feasibility, fidelity, acceptability, penetration, and sustainability.

**Conclusions:**

Implementation of SP should be understood as a system-embedded, multi-actor process in which strategies generate effects only through actor-specific enactment and the activation of mechanisms under particular contextual conditions, rather than as isolated or transferable techniques. The empirically grounded program theory developed in this study provides an analytically transferable framework for examining and guiding adaptive SP implementation and scale-up in welfare systems characterized by strong professional autonomy and decentralized governance.

## 1 Introduction

Social prescribing (SP) models are increasingly used to address complex health and social needs by linking primary care patients with community-based, non-clinical forms of support such as physical activity groups, volunteering, and peer networks (1–3). By extending care beyond the consultation by a general practitioner (GP), SP aims to bridge healthcare and civil society and address social determinants of health through cross-sector collaboration. Yet evidence on how SP models are implemented successfully across diverse organizational and welfare contexts remains limited and fragmented (3–5).

Early reviews of SP largely assess outcomes and effectiveness, treating implementation processes as contextual background rather than objects of analysis (6). They also document substantial variation in their purpose, structure, and delivery, complicating cross-study synthesis (4, 7). More recent work interprets such variation as adaptive responses to local organizational, professional, and community conditions, rather than program failure (2, 8).

Accordingly, SP is increasingly conceptualized as a context-sensitive, system-embedded model rather than a standardized model (5, 9). Implementation-focused SP reviews highlight persistent challenges, including unclear causal pathways and mechanisms, limited integration between primary care and community sectors, and weak theoretical grounding for implementation strategies (2, 3, 5, 9–11). Case studies suggest that effective implementation depends on iterative learning, boundary-spanning roles, and legitimacy-building across professional and civic domains (12–14). These findings underscore the need for program theories that move beyond cataloguing strategies to explain how strategies, actors, mechanisms and context interact to support system embedding (15). In Denmark, SP, translated as *Social Henvisning*, is an emerging policy and research field. National organizations (e.g., Danish Regions and the Danish College of General Practitioners) explore how SP models can be adapted to the Nordic welfare context through cross-sectoral collaborations between general practice, municipalities, and civil society. Early Danish studies point to implementation challenges similar to those reported internationally, including unclear role definitions, the need for structured collaboration mechanisms, and tensions between medical and community logics (16–18). Here, program theory is understood as a mechanism-based explanation of how implementation is expected to generate change, for whom, and under which contextual conditions (18).

This study develops an empirically grounded program theory for the implementation of a Danish SP model. Drawing on ethnographic fieldwork conducted within the SP *Vesterbro/Sydhavnen* project (referred to here as Southwest CPH), this study examines (1) which implementation strategies were used, (2) how strategies were distributed across key actor groups, and (3) how strategies, when performed by specific actors, activated underlying mechanisms under particularly contextual conditions to generate implementation outcomes and support system implementation.

## 2 Methods

### 2.1 The Danish SP model and setting

**Figure 1:**
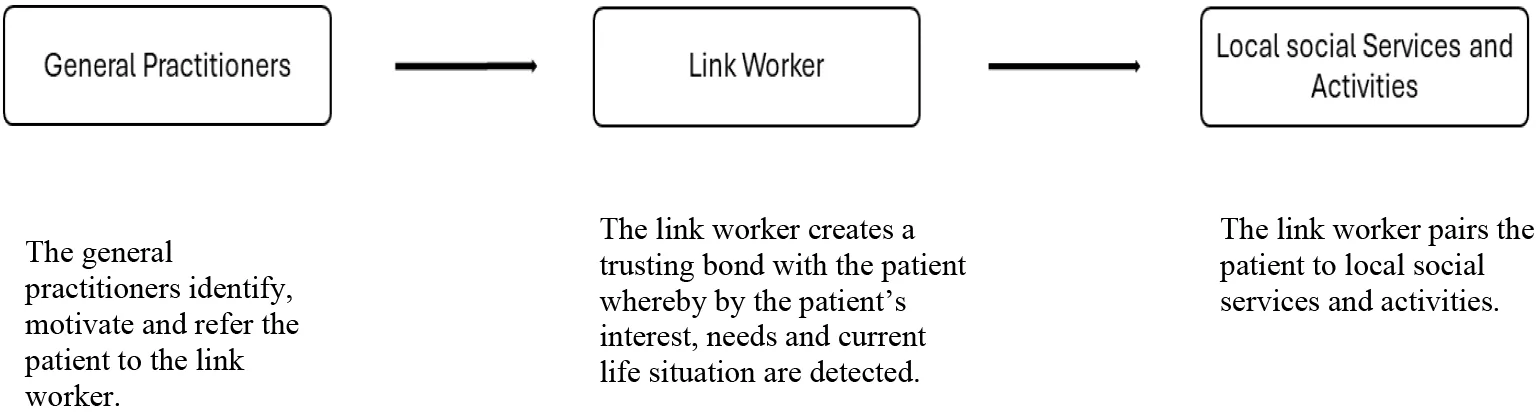
Referral pathway in the Danish SP model.

The Southwest CPH SP model was initiated and led by two GPs from Copenhagen in collaboration with the DaneAge Association, local community organizations, and a Volunteer Centre. The model consists of a referral pathway in which GPs from approximately 20 clinics refer patients to a link worker (LW), who identifies relevant community or volunteer activities in collaboration with the patient and supports engagement locally (Figure 1).

The initiative originated from two general practitioners from the Southwest Copenhagen GP cluster (Sydvestklyngen), who observed that many patients presented with social and psychosocial needs that could not be adequately addressed through conventional medical care alone. Inspired by social prescribing initiatives in the United Kingdom, particularly the Frome Model, they explored how a similar approach could be adapted to the Danish primary care and welfare context. To develop the model, they established collaborations with the DaneAge Association and a local Volunteer Centre, which contributed expertise in volunteering, community engagement, and local civil society networks. Together, these partners co-developed the referral pathway, implementation structure, and community collaborations that formed the basis of the Southwest CPH social prescribing model.

The overall evaluation program is described in the SHINE (Social Prescribing for Adults and the Elderly) research program, which comprises nine interrelated work packages (WPs) (19) (Supplementary Table S1).

This study draws on WPs 7–9 in SHINE, focusing on how implementation strategies were performed by key project actors (GPs, LWs, the Volunteer Centre, and the DaneAge Association). Patients are end-users of the model but are not responsible for its implementation and are therefore not included in the study.

#### 2.1.1 Design

This study was designed as an ethnographic field study (20), enabling an in-depth examination of how implementation strategies were performed, negotiated, and adapted during the early phase of the implementation process (from mid-2024 to mid-2025). Ethnography was well suited to capture relational and situated dynamics of implementation across organizational and professional boundaries. The study followed implementation in practice, aiming to explain implementation mechanisms rather than evaluate the effectiveness of the SP model.

#### 2.1.2 Participants

Participants were members of the Southwest CPH project group responsible for the overall implementation of the SP model: two GPs (implementation champions), three LWs, two staff from the Volunteer Centre (coordinator and manager), and one financial consultant from the DaneAge Association (*N* = 8). When referring to GPs in the results, we specifically mean the two GPs in the project group.

The LW positions were intentionally designed for individuals with a health professional background to facilitate collaboration between general practice, community organisations, and citizens referred through social prescribing. The recruited LW had backgrounds in nursing and occupational therapy and extensive experience working with individuals in socially vulnerable situations. Recruitment focused on candidates with strong relational competencies, experience in person-centred support, and the ability to navigate and collaborate across healthcare, social care, and voluntary sector settings. To support the long-term sustainability of the model, efforts are underway to establish the LW function as a permanent role within the local Volunteer Centre beyond the project period, subject to future organisational and funding arrangements.

### 2.2 Data collection

Participant observations were conducted throughout the first year of the implementation of the SP model and documented in field notes. Observations covered coordination meetings, GP cluster meetings, local networking events, and project workshops. Additional documentary materials (e.g., emails, educational materials, and project reports) were collected to map the strategies used and their interconnections.

Data collection was conducted by the first and second authors (JWK and LSO), both of whom have strong qualitative research experience. JWK is an associate professor in implementation research and served as principal investigator for the qualitative evaluation and analysis. LSO is a public health research assistant with primary responsibility for data collection and contributions to the data analysis. Neither researcher had previously conducted research in general practice, rendering this field new to both. Reflexive field notes and regular analytic discussions were used to support interpretive rigour and credibility.

As researchers embedded within the broader SHINE research programme, we had prolonged access to the implementation process and regular interactions with key project actors. This position provided valuable insight into ongoing implementation activities and facilitated trust and openness during fieldwork. At the same time, we were aware that our presence and sustained engagement could influence participants’ reflections and discussions about implementation. We therefore approached data collection and analysis reflexively, continuously examining how our professional backgrounds in implementation research and public health, our assumptions, and our relationships with participants might shape data production and interpretation. Analytic discussions within the research team were used to challenge emerging interpretations and strengthen reflexive awareness throughout the study.

To strengthen credibility and explanatory depth, we conducted two iterative validation rounds with all project members. The research team circulated preliminary findings (e.g., the strategy list and emerging assumptions about mechanisms and outcomes) for verification and elaboration. This process combined the principles of member validation (21, 22) with the theory elicitation techniques from realist evaluation (15, 23). These rounds functioned as analytical dialogue, rather than data correction and supported refinement of the emerging program theory.

### 2.3 Data analysis: five analytical steps

The analysis comprised five analytical steps to distinguish empirical description, interpretive analysis, and theoretical synthesis: (1) mapping implementation strategies; (2) identifying roles across key actors; (3) analysing strategies according to systemic logic; (4) identifying actor logics and mechanisms; and (5) developing a program theory. The analysis followed an abductive analytical logic (24), iterating between empirical material and theoretical concepts. Realist evaluation (15), ethnography (20), and systems theory (25) informed the analysis across all steps.

In the first analytical step, all field notes (approximately 182 pages) were read by JWK in full to gain an overall understanding of the empirical material. This initial familiarization enabled systematic empirical mapping and categorization of implementation strategies, focusing on what was done in practice. Then, JWK coded the strategies using the ERIC taxonomy (26) and compiled a strategy matrix (Supplementary Table S2). The ERIC mapping was used analytically to catalogue observed activities, not as a prescriptive checklist; explanatory insight lies in how strategies, when performed by specific actors, activate mechanisms under particular contextual conditions. The matrix was subsequently reviewed by MBH and LSO for verification; disagreements were resolved through discussion until consensus was reached regarding whether the strategies should be mapped to the same or to a different ERIC category.

In the second step, we analysed how implementation strategies were distributed across actor groups and how this distribution translated into differentiated actor roles (Table 1 and Supplementary Table S3). This step identified patterned role configurations based on observed activities. We drew on Hasse's concept of cultural models (27), understood as professionally and organizationally embedded orientations shaping judgement, meaning-making, and action, to analyse how the actors made sense of their roles and prioritized particular strategies.

**Table 1:** Distribution of implementation strategy activity across the ERIC clusters and actor groups.

ERIC cluster	General practitioners	Link workers	Volunteer centre	DaneAge Association
Dissemination strategies	●●●	●●	●●	●●
Policy strategies	●●●	●	●	●●●
Stakeholder interrelationships strategies	●●	●●●	●●●	●
Training and education strategies	●	●●●	●●	●
Change infrastructure strategies	●●●	●●	●	●
Evaluative & iterative strategies	●●	●●●	●	●

●●●, high activity; ●●, moderate activity; ●, limited activity.

In the third step, we analysed how implementation strategies and actor functions interacted across settings and over time, focusing on patterns of interaction and coordination rather than isolated actions. Drawing on systems theory and the notion of “events in system” (25), we analysed implementation strategies as system events that disturbed and reshaped organizational routines, relationships, and infrastructures across clinical, community, and governance settings. This step examined how strategies were combined, negotiated, and adjusted through ongoing coordination across settings, and how their mechanisms of action emerged through alignment and mutual reinforcement rather than through linear causal chains.

In the fourth step, we analysed actor logics and associated mechanisms by examining how actors made sense of their implementation work and how they justified and prioritized strategies. Actor logics were analysed as culturally and professionally embedded orientations through the concept of cultural models (27), linked to mechanisms operating under specific contextual conditions, resulting in a mapping of dominant actor logics and mechanisms across actor groups.

In the fifth analytical step, we synthesized findings into an empirically grounded program theory explaining how implementation strategies were expected to generate outcomes, for whom, and under which contextual conditions. The synthesis linked strategies, actor functions, cross-cutting mechanism families, contextual conditions, and implementation outcomes, drawing on Proctor et al.'s taxonomy (28).

### 2.4 Ethical considerations

The study was conducted in accordance with the ethical principles of the Declaration of Helsinki and the Danish Code of Conduct for Research Integrity (29). All data were handled in compliance with the General Data Protection Regulation and registered with the Capital Region of Denmark. Written informed consent was obtained from all participants. The study was approved by the Capital Region of Denmark on behalf of the Danish Data Protection Agency (p-2024–17318). A waiver of ethical approval was granted by the Regional Committee on Health Research Ethics (journal no. F-25028691), which determined that the study was not subject to notification requirements under Danish legislation because it was based on qualitative observations and document analysis and because the social prescribing intervention formed part of routine clinical practice. The effectiveness evaluation was registered prospectively at ClinicalTrials.gov (NCT07029334).

In addition to formal regulatory approval, ethical considerations were addressed continuously through a situational and reflexive approach inherent to ethnographic research (30). Following Hammersley and Atkinson (20), we recognize that researchers are unavoidably implicated in the social worlds that they study, and that ethical issues emerge through ongoing interactions rather than being fully resolved in advance. During prolonged participant observation across multiple contexts, we sought to maintain analytical distance by positioning ourselves outside the project group. However, sustained engagement meant that we were occasionally asked to clarify factual matters or reflect on emerging issues, requiring ongoing ethical judgement regarding role boundaries. These situations were addressed through reflexive practice, including field notes documenting role negotiations and their potential influence on data production.

Throughout the study, attention was paid to participant vulnerability, power relations, voluntariness, and confidentiality, and ethical sensitivity was exercised as a situated balance between engagement and analytical distance.

## 3 Results

### 3.1 Mapping of implementation strategies

In the first analytical step, we identified a broad set of implementation strategies used during the first year of the implementation process. Strategies spanned six ERIC clusters: dissemination, policy strategies, develop stakeholder interrelationships, train and educate stakeholders, infrastructure change, and evaluative and iterative strategies (26).

To improve readability, the full ERIC strategy matrix is provided in Supplementary Table S2 and summarized in Table 1. The key distributed pattern can be summarized as follows: implementation work was distributed across four actor groups (GPs, LWs, the Volunteer Centre, and the DaneAge Association), with distinct emphases that formed the empirical basis for subsequent analysis of actor functions and mechanisms.

Overall, strategies were used by all four actor groups, but the number and type of strategies varied across clusters. GPs were involved in a higher number of strategies within dissemination, policy strategies, and infrastructure change. LWs were involved in a higher number of strategies related to training and education, stakeholder interrelationships, and evaluative and iterative activities. The Volunteer Centre was especially involved in strategies related to stakeholder interrelationships, training and education; the DaneAge Association was involved in strategies related to dissemination and policy:

At a leadership meeting, the DaneAge representative and the GPs discuss which political parties in the Danish Parliament they have each planned meetings with to promote SP and highlight the health-related and social perspectives of integrating SP at a national level. [field notes, October 2025]

This descriptive mapping provides the empirical foundation for the subsequent analysis of actor functions and role distribution.

### 3.2 Identifying roles across key actors

The second analytical step examined how the observed distribution of implementation strategies translated into differentiated actor roles within the implementation system. Rather than treating roles as formally assigned positions, the analysis focused on how actor functions emerged through repeated patterns of activity across settings and over time. Fieldwork observations showed a differentiated but complementary pattern of actor roles. An observation from a local network meeting illustrates how these roles were performed in practice:

During the meeting, local organizations directed nearly all practical questions to the LWs, who explained referral processes, participant needs, and support options. The Volunteer Centre facilitated the session, set the agenda, and encouraged cross-organizational networking. The DaneAge representative contributed mainly to issues involving volunteering and national strategies. GPs were frequently referenced as initiators of the SP pathway, but were not present and did not participate in implementation discussions. [field note, May 2025]

This observation illustrates how implementation work was distributed across actors *in situ*. Although GPs initiated the SP pathway and remained central to strategic and organizational decisions, their role in day-to-day implementation activities was limited. Instead, LWs occupied a central position in operational coordination, acting as primary points of contact for both community organizations and clinical actors.

Across the empirical material, LWs consistently performed activities that connected general practice with community and voluntary sector settings. Through training activities, follow-up, and ongoing dialogue with local organizations, they translated referral criteria, participant needs, and organizational expectations across sectoral boundaries. In this way, LWs functioned as coordinators of implementation work across settings rather than as isolated service providers. This was addressed at a joint meeting in the project group. An LW expressed:

We’ve had very positive experiences holding lunchtime meetings at GP clinics. The informal setting creates an open dialogue where we can share our knowledge about SP while also listening to their needs and perspectives. It has proven to be an effective way to build interest and support. [LW 1, June 2025]

The Volunteer Centre played a complementary role by facilitating local networking, convening meetings, and supporting collaboration among community organizations and volunteers. Their activities focused on creating and maintaining local infrastructures that enabled the LWs’ coordination work and supported the practical inclusion of referred participants. The manager at the Volunteer Centre expressed:

LW 3 has done an outstanding job reaching out to both new and existing civil society organizations. Her visits have not only strengthened collaboration but also given us a much deeper understanding of the challenges and needs within civil society when receiving citizens referred through SP. [manager, September 2025]

The DaneAge Association operated primarily at a strategic and national level, contributing through policy-oriented activities, public dissemination, and coalition-building. Rather than engaging in local operational tasks, their role centred on strengthening political legitimacy, aligning the SP model with broader welfare agendas, and supporting longer-term sustainability.

These patterns indicated a multi-actor implementation structure characterized by complementary actor roles. Implementation was sustained not by a single coordinating actor but through the alignment of strategic leadership, operational coordination, local network facilitation, and national advocacy across organizational levels. The distribution of the actor roles identified in the analysis is summarized in Table 2. These patterns also describe how implementation work was empirically distributed across actors and organizational levels. The role analysis established what different actors did within the implementation system. The next analytical step shifts the focus from actor roles to how the implementation activities interact systemically across contexts and over time.

**Table 2:** Distribution of roles.

Actors	Number of clusters with activity	Characteristics
General practitioners	Active in almost all clusters (5/6)	Dominant in policy, dissemination, and change infrastructure; relatively little involvement in direct training and feedback (these activities are mainly carried out by LWs)
Link workers	Very broad coverage; particularly in training, stakeholder relationships, and evaluative strategies	Function as link workers and facilitators
The Volunteer Centre	Most active in dissemination, stakeholder interrelationships, and training	Acts as a local partner for networking and community engagement
The DaneAge Association	Mainly active in policy and dissemination	Serves as a national legitimizing and coalition-building actor

### 3.3 Analysing strategies according to systemic logic

The third analytical step examined how implementation strategies interacted across settings and over time, and how these interactions shaped the implementation process at a system level. The preceding role analysis established what different actors did within the implementation system; this step focused on how these activities were combined, coordinated, and mutually adjusted across clinical, community, and governance contexts. Across the empirical material, strategies were rarely performed as isolated actions; instead, they functioned as interdependent system events, and their effects emerged through ongoing interaction.

An illustrative example of such system-level interaction was observed during a cluster meeting with GPs. Cluster meetings are recurring forums where GPs from multiple clinics coordinate care, exchange experiences, and discuss the implementation of new initiatives and quality improvement activities:

During a cluster meeting with about 40 GPs, a GP implementation champion presented recent updates to the SP model, including the expansion to patients aged 18 + years and the hiring of a new coordinator. The presentation prompted a series of practical questions from the GPs: whether younger patients would require new activities, whether smaller referral cards could be produced, whether non-Danish-speaking patients could be included, and whether a QR code could support easier access to information. [field notes, March 2025]

Rather than functioning as one-way dissemination, this interaction created a dialogical space in which dissemination, infrastructural considerations, and governance questions were negotiated simultaneously. Inclusion criteria, referral routines, and supporting tools were adjusted through collective discussion, illustrating how strategies operated through interaction rather than sequential implementation. After the meeting, the presenting GP noted that “more was already happening” after the appointment of the coordinator, indicating that the introduction of new organizational elements interacted with existing strategies to enhance coordination and responsiveness within the system.

Similar interactional dynamics were observed across other implementation arenas. In project and leadership meetings, discussions of documentation indicators, governance responsibilities, and clinical integration unfolded concurrently, reflecting how organizational, practical, and policy-related concerns were continuously aligned rather than addressed in discrete phases:

During the discussion, participants moved freely between practical concerns about referral routines, questions on documentation indicators, and broader governance responsibilities. Rather than separating strategic, clinical, and organizational issues, these were addressed in the same conversational flow. At one point, a GP remarked that “we are adjusting as we speak”, capturing how implementation unfolded through ongoing negotiation rather than through predefined phases. [field note, November 2025]

These interactions highlight how implementation progressed through iterative negotiation across professional and organizational boundaries.

Interactions across healthcare and community contexts further illustrated this systemic logic. Building on the coordinating role of LWs described in the role analysis above, activities such as training, follow-up, and stakeholder engagement functioned systemically by maintaining feedback loops between general practice, community organizations, and participants: LW 2 explained:

We stay in regular contact with the GPs to follow up on how the referred patients are doing. That ongoing dialogue helps us adjust our support and ensures that the process doesn't stop at the point of referral. [LW 2, September 2026]

These feedback loops supported learning and adaptation across settings, enabling the SP model to be translated into workable local practices rather than standardized uniformly.

Similarly, the network-building activities facilitated by the Volunteer Centre, described in detail above, operated at a system level by expanding local implementation capacity. By convening community organizations and enabling coordination and formation of partnerships, these activities strengthened the infrastructural conditions under which referral pathways and follow-up practices could be sustained across contexts.

At a national level, policy advocacy and public visibility efforts, primarily undertaken by the DaneAge Association, interacted with local implementation work by shaping political legitimacy and strategic positioning. These activities influenced the broader conditions under which actors prioritized and sustained implementation efforts, demonstrating how governance-level strategies formed an integral part of the overall implementation system.

This systemic analysis demonstrates that implementation of the SP model unfolded through interdependent interactions across multiple system levels. Strategies did not generate effects independently, but through their alignment and mutual reinforcement across clinical, community, and policy contexts. However, although this analysis clarifies how strategies interact across the system, it does not yet explain why actors prioritized particular actions or how they made sense of their roles in practice. To address this, the next analytical step turns to actor logics and the mechanisms through which strategies were interpreted and enacted.

### 3.4 From systemic logic to actor logics

In the fourth analytical step, actor logics and mechanisms are treated as analytically distinct but closely related concepts. Actor logics refer to the culturally embedded orientations through which actors interpret their roles, priorities, and responsibilities within the implementation process.

Mechanisms, in contrast, denote the causal processes that are triggered when strategies performed by actors interact with specific contextual conditions. Actor logics thus inform which strategies are pursued and how they are performed, whereas mechanisms explain how these strategies are assumed to generate effects in practice.

Drawing on Hasse's concept of cultural models (27), actor logics are understood as professional and organizational embedded orientations comprising professional routines, interpretive frameworks, and normative expectations that guide what actors attend to and how they define appropriate action (31). From this perspective, implementation is not only a system-level process but also a situated practice shaped by collective actor-specific sense-making.

Empirically, these actor logics became visible in how different groups justified and prioritized their implementation activities. For example, both GPs emphasized the importance of aligning SP with established clinical standards and workflows. As one GP noted: “If we are to prioritize SP in a busy clinic, it has to make clinical sense. We need to know that it works and that it fits with our professional standards.” Here, SP was framed through a cultural model centred on professional legitimacy, standardization, and efficiency, directing attention towards guidelines, documentation practices, and IT integration.

At the national level, representatives from the DaneAge Association articulated a political and advocacy-oriented logic. As one representative stated: “To secure sustainability, SP must be recognized politically. We need alliances and policy backing.” Here, implementation was framed through coalition-building and strengthening the mandate, reflecting a cultural model in which durability depends on political legitimacy and institutional anchoring.

Based on a cross-case analysis of fieldwork observations and interview material, we identified dominant actor logics and associated mechanisms across the four key actor groups involved in implementing the SP model. This analysis does not yet constitute a program theory; rather, it provides an analytical mapping of how different actors oriented themselves within the implementation process and how recurring rationales and mechanisms shaped their engagement and prioritization of implementation activities.

Table 3 summarizes the dominant actor logics and associated mechanisms identified across the four key actor groups involved in implementing the SP model, along with their expected implementation outcomes. The table illustrates how actors’ professional and organizational orientations informed distinct rationales for action and were linked to different dominant mechanisms within the implementation system.

**Table 3:** Actor logics and dominant mechanisms shaping engagement in SP implementation.

Actors	Dominant mechanisms	Rational choices/justifications	Expected implementation outcomes
General practitioners	Professional legitimacy, standardization, efficiency	Strategies are selected to align SP with clinical routines and professional identity (e.g., guidelines, articles, IT integration)	Adoption, fidelity, feasibility
Link workers	Relational trust, mutual learning, sense-making	Practices emphasize trust-building and adaptive learning across clinical and community contexts	Acceptability, penetration, sustainability
The Volunteer Centre	Community embeddedness, network weaving	Actions focus on mobilizing local engagement and strengthening cross-sectoral collaboration	Reach, acceptability
The DaneAge Association	Political legitimacy, advocacy, coalition-building	Efforts target policy influence and coalition formation to strengthen the mandate and legitimacy	Mandate change, sustainability

Although this actor logic analysis clarifies how actors made sense of implementation work within particular contexts, it remains analytically fragmented across actor groups. Therefore, the final analytical step synthesizes these actor-specific logics and mechanisms into a program theory explaining how implementation strategies generate outcomes through underlying mechanisms operating across actors and settings under specific contextual conditions.

### 3.5 Developing a program theory

Building on the actor-level analysis, this section presents an empirically grounded program theory, explaining how implementation strategies generate change through mechanisms operating across actors and settings under specific contextual conditions. Whereas the previous section focused on actor-specific logics and mechanisms, the program theory synthesizes these findings into cross-cutting mechanism families operating at the system level.

The importance of context for mechanism activation was evident in general practice, where one clinical staff member noted:

We really want to help with the project, but we simply have so much to do that we often forget to refer, even though we think it's a good idea. [field note, October 2025]

This quote illustrates how workload pressures and competing demands can inhibit otherwise relevant mechanisms. Thus, implementation depends not only on the presence of strategies, but on whether they resonate with local practices and activate mechanisms under favourable contextual conditions.

Across the empirical material, five cross-cutting mechanism groups were identified through an analytic synthesis of actor logics (Table 3), fieldwork observations, and interview data:

1. **Legitimacy**, activated when dissemination, championing, and policy-related activities enhanced professional and political credibility (e.g., through presentations from GP champions in cluster meetings);
2. **Standardization and normalization**, activated through integration of SP into clinical workflows (e.g., referral guidelines, IT systems, and documentation routines);
3. **Relational sense-making and trust-building**, activated through ongoing dialogue and follow-up across healthcare and community settings;
4. **Network weaving and coalition formation**, activated through mobilization of actors across organizational and sectoral boundaries, (e.g., in network meetings at the Volunteer Centre where new collaborations were initiated);
5. **Knowledge visibility and mobilization** activated when information about SP, referral pathways, and support options was made visible and actionable for clinicians, community organizations, and patients (e.g., through meetings, materials, and direct interaction).

These mechanism families abstract and integrate actor-specific mechanisms at a system level, capturing recurrent patterns of how strategies generate effects across settings, regardless of the initiating actor.

**Figure 2:**
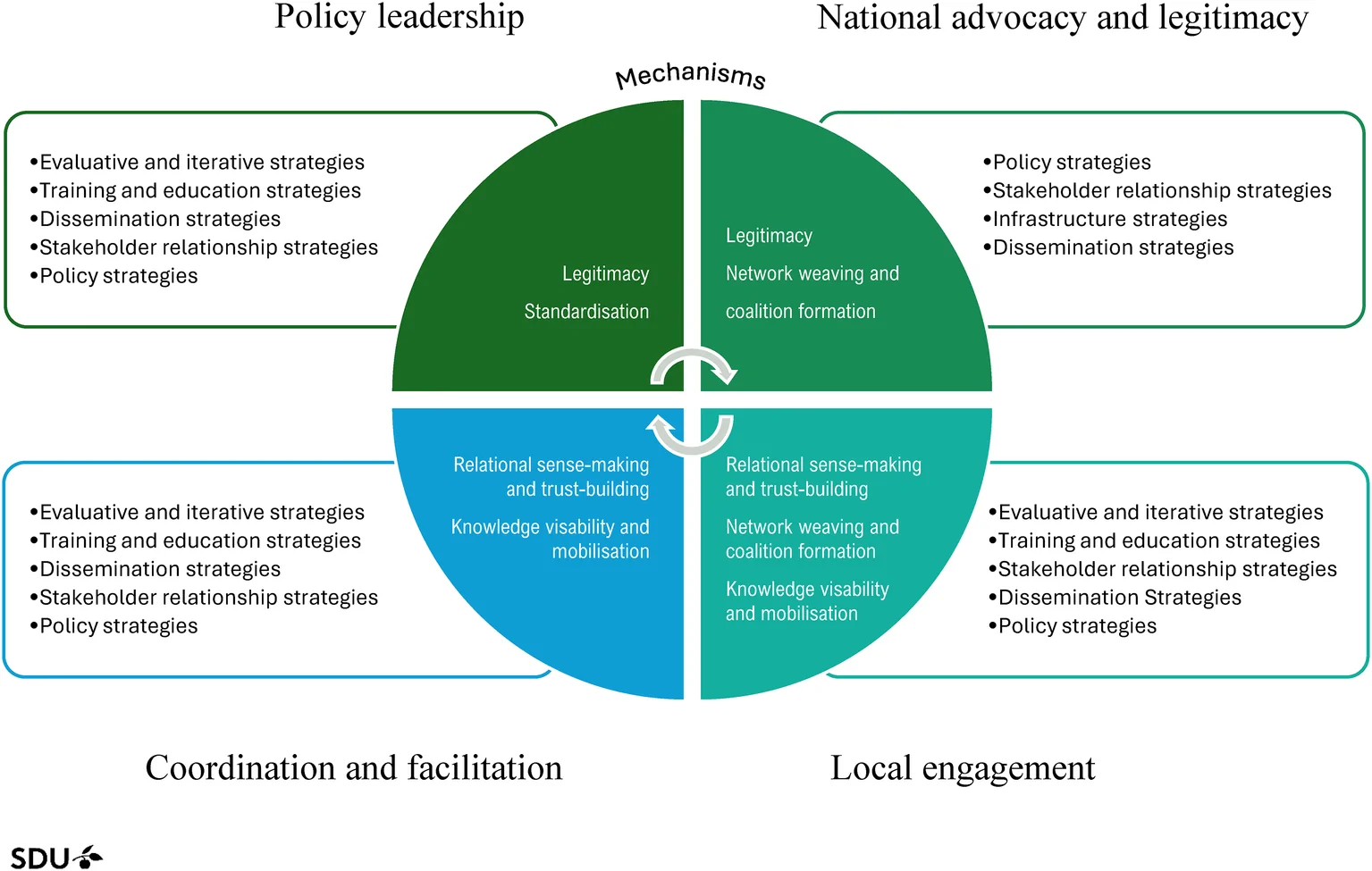
The program theory.

Each implementation strategy was subsequently linked to its expected mechanism(s), relevant contextual preconditions (e.g., workload, organizational capacity, policy alignment), and analysed in relation to proximal, intermediate, and distal implementation outcomes consistent with Proctor et al.'s (28) taxonomy. This synthesis marks the analytical shift from a structural mapping of strategies to a mechanistic explanation of how implementation strategies generate change through actors’ situated practices. Figure 2 presents the resulting program theory, synthesizing strategies, mechanisms, contextual conditions, and implementation outcomes.

To consolidate the causal logic, four system-level propositions summarize how combinations of strategies and mechanisms are expected to generate implementation outcomes:

1. If dissemination and championing strategies strengthen professional and political legitimacy, adoption and penetration will increase across sectors.
2. If infrastructural and educational strategies support standardization and integration into everyday workflows, fidelity and feasibility will be strengthened.
3. If collaborative practices support relational sense-making and trust-building across sectors, acceptability and appropriateness will improve.
4. If coalition-building, policy alignment, and iterative learning processes are sustained across institutional boundaries, long-term sustainability and system embedding will follow.

These propositions bring together the core assumptions of the program theory linking empirically observed strategies and mechanisms with expected implementation outcomes.

## 4 Discussion

This study contributes to the SP literature by developing an empirically grounded program theory explaining how implementation strategies generate change through interactions between actors, mechanisms and contextual conditions within a complex adaptive system. SP is increasingly promoted as a means to address non-medical determinants of health, but existing research highlights substantial variation in implementation processes and outcomes across settings, alongside limited theoretical explanation for this variation (6, 10, 32, 33). Recent reviews further emphasize that much of the SP evidence base remains descriptive and under-theorized, particularly with regard to implementation processes (34). By conceptualizing implementation strategies as system events rather than discrete and transferable techniques, the findings respond directly to calls for more theory-driven and mechanism-based accounts of SP implementation that deal with context, actor roles and organizational dynamics (35, 36).

Accordingly, the program theory shifts attention away from checklist-oriented uses of implementation strategies towards explaining how strategies operate through actor-specific performance and context-dependent mechanisms. Rather than asking whether SP works, this study demonstrates how implementation strategies operate differently depending on who performs them, how they are performed, and how they resonate within existing professional routines, organizational infrastructures and institutional logics. This perspective aligns with systematic reviews conceptualizing SP as a complex service innovation composed of interdependent relational, organizational and political elements (3, 35, 36). The study thus advances the SP field by moving beyond descriptive accounts of contextual influence towards explanatory insight into system-level implementation dynamics.

A central contribution of the program theory is its differentiated yet complementary account of implementation work across actors. Much of the international SP literature positions LWs as the primary drivers of implementation (3, 32). This study confirms the importance of LWs in spanning boundaries, facilitating relational sense-making, trust-building and feedback loops, but it also demonstrates that system-level embedding of SP depends on coordinated role differentiation across multiple actors. In particular, GPs played a decisive role in building legitimacy, infrastructural anchoring, and policy alignment, echoing Danish primary care research showing that GPs rarely address issues, such as loneliness, systematically without organizational support (33, 37, 38).

The involvement of the Volunteer Centre and the DaneAge Association highlights dimensions that remain under-theorized in much SP research. Although the capacity, connectivity and sustainability of the voluntary and community sector are widely recognized as preconditions for the feasibility and long-term impact of SP, they are often treated as contextual background rather than active components of implementation (13, 35). By explicitly analysing how civic and national-level actors contributed to strengthening local infrastructures and political legitimacy, this study extends prevailing SP models and underscores that implementation involves institutional and coalition-building work alongside relational and clinical practices.

Across the findings, implementation success hinges on whether strategies activate specific mechanism families under favourable contextual conditions. This addresses a recurrent limitation in SP evaluations, which often report mixed or modest effects without adequately explaining how workload pressures, competing priorities, and resource constraints shape implementation outcomes (3, 38).

The empirical material illustrates how strategies were interpreted and performed differently across actors and settings, and how the assumed effects of the strategies depended on whether they resonated with existing professional routines and organizational arrangements. For example, dissemination and championing strategies were associated with increased attention to and uptake of SP when they enhanced professional and political legitimacy, whereas infrastructural strategies were assumed to support fidelity and feasibility primarily when aligned with everyday clinical workflows.

These findings support a program-theoretical interpretation in which the contribution of implementation strategies cannot be understood in isolation, but as contingent on contextual conditions that enable or constrain the activation of relevant mechanisms. In this sense, the study aligns with the existing literature, suggesting that the presence of a strategy alone is insufficient to explain variation in implementation outcomes and highlighting the need for subsequent evaluative research to examine whether and how these mechanisms translate into improved implementation outcomes in practice (35).

By synthesizing actor-specific logics into cross-cutting mechanism families, the study offers a middle-range program theory that balances contextual specificity with analytical abstraction. This responds to longstanding critiques of the SP evidence base as fragmented, heterogeneous and under-theorized (6). The four system-level propositions articulate how combinations of strategies and mechanisms are expected to generate key implementation outcomes, including adoption, acceptability, feasibility and sustainability. Thus, the program theory provides an analytically transferable framework to inform future research and guide adaptive implementation of SP in welfare contexts characterized by strong professional autonomy and decentralized governance (34).

### 4.1 Operational implications for implementation and scale-up

The program theory developed in this study can be operationalized by translating its core mechanisms into concrete implementation functions at the clinical, organizational, and system levels. For general practice, the mechanisms of legitimacy and normalization imply that SP needs to be embedded at specific decision points within everyday clinical workflows. Operationally, this entails defining when SP is considered during consultations (e.g., in relation to recurrent attendance, psychosocial distress, or functional decline), what constitutes a minimum viable referral (e.g., problem orientation, patient preferences, and contact information), and how responsibilities are distributed between GPs, practice staff, and LWs.

The mechanisms of relational sense-making and trust-building further imply that implementation requires structured feedback loops between GPs, LWs, and community actors. In operational terms, this includes routine follow-up communication that confirms receipt of referrals, provides brief status updates, and enables practices to recalibrate referral behaviour over time.

At the organizational and system levels, the mechanisms of standardization, network weaving, and coalition formation indicate a set of core infrastructural functions necessary for regional or national implementation. These include integration of referral templates into electronic health record systems, sufficient and predictable capacity among LWs, maintaining an overview of community resources, and formalized cross-sector coordination structures that distribute implementation work across clinical, community, and policy domains.

These operational functions should be understood as expressions of the underlying program theory rather than as prescriptive components. Although their concrete form may vary across settings, their presence constitutes a set of core implementation requirements through which the identified mechanisms are expected to generate adoption, feasibility, fidelity, and sustainability in welfare systems characterized by high professional autonomy and decentralized governance.

### 4.2 Limitations

Several limitations should be acknowledged. The study is based on a single case in a specific Danish urban context characterized by strong implementation champions and established cross-sectoral relationships. Although this enabled in-depth analysis of implementation processes, it may limit the transferability to contexts with weaker institutional support, fewer resources or less mature community infrastructures. The program theory should therefore be understood as analytically transferable rather than universally generalizable.

The empirical material primarily reflects the perspectives and practices of central implementation actors. Patients and front-line clinical staff beyond the core project group were not included. Although this focus was theoretically justified given the study's emphasis on implementation work rather than use of the model, it may under-represent how implementation strategies are experienced, resisted or adapted at the point of care. Future research could extend the program theory by incorporating patient and front-line perspectives.

The focus of this study was to develop a program theory for the implementation of SP and thereby contribute to a theoretical explanation of SP implementation. The study does not allow us to determine whether the identified strategies led to the expected outcomes or whether they strengthened implementation. Future research is therefore needed to explore in depth how, for whom, and under which circumstances the selected implementation strategies activate the identified mechanisms, and whether these, in turn, result in improved implementation of SP (40).

The close and prolonged engagement inherent to ethnographic research raises the possibility that the researchers’ presence influenced implementation processes. Despite sustained reflexive practices and efforts to maintain analytical distance, occasional involvement in discussions may have shaped sense-making processes and learning-oriented dynamics (39). Rather than viewing this solely as bias, it may also highlight how research activities can function as implementation events, warranting further theoretical and methodological reflection.

## 5 Conclusion

This study shows that the implementation of SP depends not on isolated strategies, but on how strategies are performed by different actors and how they activate mechanisms under specific contextual conditions. By developing an empirically grounded, mechanism-based program theory, the study advances implementation science by clarifying the causal logic through which strategies, actor functions, mechanisms, and context interact in a complex, multi-actor system.

The findings demonstrate that implementation work is distributed across complementary actor roles, and that successful incorporation in the system relies on relational coordination, legitimacy-building, and alignment across clinical, community, and policy domains. Rather than assuming that strategies operate uniformly, the study shows how different actors hold and enact distinct assumptions about which strategies are meaningful and how they are expected to generate change under particular conditions.

For policy-makers and practitioners, the program theory highlights that investing in infrastructural supports, boundary-spanning roles, and coalition-building processes is as critical as promoting individual implementation strategies. For researchers, the study offers an analytically transferable framework for examining how implementation strategies operate through mechanisms across contexts, contributing to a more explanatory and system-oriented evidence base for implementation of SP.

## Data Availability

The dataset generated and analysed during the current study is not publicly available because it contains potentially identifying or sensitive information that could compromise the privacy of the respondents, in accordance with regulations set out by the Danish Data Protection Agency. However, it is available from the corresponding author upon reasonable request.
