# Supplementary material for: Developing a program theory for implementing a Danish social prescribing model: an ethnographic study
Source: Front Health Serv. 2026 Aug 11;6:1815178. doi: 10.3389/frhs.2026.1815178 (PMC13503515; doi:10.3389/frhs.2026.1815178)
Supplement: Supplementary file 1 [file Table1.docx]

Supplementary Material

# Supplementary Table 1. The social prescribing research program

|  |  | **Methods** |
| --- | --- | --- |
| **Intervention components** | | |
| **WP 1** | Determinant and acceptability study | Ethnographic field work based on selected consultations with general practitioners; semi-structured interviews with patients conducted shortly after referral and again approximately 6 months later. In addition, semi-structured interviews were held with the link worker during the pilot period and with general practitioners 9 months after the initial patient consultations. |
| **WP 2** | Identification of patient profiles for referral until matching with voluntary services | The study applied a quantitative method to identify patient profiles in the SydVest Cluster via data from medical records, the health register and T-ILS/UCLA-3 for loneliness. Descriptive statistics were used to uncover profiles and variation in referral practices. |
| **WP 3** | The link workers’ competencies and mechanisms in collaboration with practitioners and patients | Ethnographic field study where the link worker was observed for approximately 1 month. Followed up by semi-structured interviews. |
| **WP 4** | The suitability of the match process in relation to the target group | The study applied a realistic evaluation approach to strengthen the program theory of social prescribing to voluntary services. Ethnographic fieldwork explored patients’ encounters with the link worker and voluntary services, supplemented by interviews conducted after the initial meeting and again 3 months later. |
| **WP 5** | The effect of the intervention on healthcare utilization, loneliness and quality of life | Individual-level data on medication, healthcare contacts and participation in social services were collected from health register records from the link worker and questionnaires. Changes were compared with a demographically matched control group from the SydVest Cluster and analysed over a 9-month follow-up period. |
| **WP 6** | Combined process evaluation | Data from WP 1–8 were integrated using a convergent mixed-method design, combining qualitative and quantitative data. The aim was to identify key factors for effective implementation and long-term sustainability of social prescribing, and to develop recommendations for optimizing the matching and integration of civil society services across diverse patient profiles. |
| **Implementation components** | | |
| **WP 7** | Mapping implementation strategies | Maps and categories of implementation strategies used by general practitioners, link workers and the Volunteer Centre during pre-implementation. An initial program theory was developed linking strategies to expected outcomes through systematic mapping and participants’ input. |
| **WP 8** | Ethnographic field study | An ethnographic field study mapped the use of selected and changed implementation strategies, followed up with semi-structured interviews for justifications for choosing new strategies. |
| **WP 9** | Realistic evaluation | Data from the intervention in the pilot project was used and supplemented with interviews with practitioners, the link worker, and actors from the voluntary services at the end of the pilot period. In addition, quantitative questionnaires and data analysis were sent out to assess which actors (practitioners, types of clinics) had the greatest success with the implementation. |

WP, Work Package.

# Supplementary Table 2. The strategy matrix

| Distinct strategies (ERIC) | Precondition for mechanism activation | Moderators | Mechanisms | Short-term outcomes | Medium-term outcomes | Long-term outcomes |
| --- | --- | --- | --- | --- | --- | --- |
| Dissemination strategies | | | | | | |
| Newspaper articles: use mass media | Activation of this strategy requires access to relevant media outlets and journalists who can help communicate the link worker initiative | The effectiveness is moderated by the reach and credibility of the media coverage | Public and professional attention to the initiative | Awareness among professionals and citizens | Actors show interest in engaging with the Bridge Builder; visibility and credibility facilitate greater adoption and penetration of the link worker model within professional practice and service delivery | Sustainability of the implementation by maintaining ongoing awareness and legitimacy among key stakeholders |
| Website: Use mass media, public visibility | A well-functioning and regularly updated website is a key precondition for this strategy | Its effect is moderated by user experience, accessibility, and search engine visibility | Easily accessible information, materials, and contact options, thereby supporting transparency and consistent communication about the link worker initiative. | Awareness and access to relevant knowledge | Increase in inquiries from citizens, professionals, and potential collaborators | Visibility, trust, and engagement with the initiative, thereby facilitating adoption, reach, and ultimately the sustainability of the implementation by ensuring ongoing access to accurate, up-to-date resources |
| Develop new materials for an extended target group 18+ years, including a new link worker: Develop and distribute educational materials | This strategy requires updated and accessible materials, along with effective distribution channels | Its impact is moderated by the quality of the brochure design, clarity of language, and appropriateness of audience targeting, particularly as the initiative expands to include adults ≥18 years | The mechanism operates through increased visibility of the link worker service and clear communication about how to engage with it | In the short term, this enhances awareness and understanding of the service among the target audience | In the medium term, it leads to more inquiries and engagement, particularly from the newly included 18+ years demographic | Over time, this targeted communication supports greater inclusion and normalization of the link worker role across diverse user groups, thereby promoting adoption, acceptability, and sustainability of the service in different community contexts |
| Request for a video introducing the link worker (not yet developed): Develop and distribute educational materials (video as an educational tool) | For this strategy to be activated, the introductory video must first be produced, and appropriate dissemination channels, such as social media platforms and the project website, must be established | The effectiveness of the strategy is moderated by the quality, clarity, and reach of the video | The mechanism relies on visual and narrative communication to convey the purpose, values, and practical role of the link worker; by presenting relatable stories or real-life examples, the video helps audiences connect emotionally and cognitively with the concept | In the short term, this increases engagement and understanding among both professionals and potential service users | In the medium term, it builds trust, motivation, and perceived relevance of the link worker model | Over time, these effects support stronger stakeholder commitment and contribute to sustained use of the approach, leading to improved adoption, acceptability, and fidelity in implementation |
| E-mails from the implementation champion (a general practitioner [GP]): Conduct educational outreach visits | This strategy presupposes updated and well-maintained contact lists, as well as access to relevant professional networks | The effectiveness is moderated by the tone, clarity, and personalization of the communication | The mechanism involves direct, relational communication that fosters trust, engagement, and collaboration among stakeholders | In the short term, the e-mails generate awareness of the link worker initiative and prompt responses from recipients | In the medium term, they help establish and strengthen professional relationships, promoting shared ownership and alignment around the link worker approach | Over time, these trusted communication channels support continuous engagement and collaboration across sectors, thereby reinforcing the acceptability, adoption, and sustainability of the implementation process |
| Articles in the Occupational Therapy Magazine or other professional journals and newspapers: Use mass media | This strategy requires access to relevant professional and medical publications capable of reaching practitioners, researchers, and decision-makers | Its effect is moderated by the credibility, reputation, and professional relevance of the chosen media outlets | The mechanism functions through the creation of professional legitimacy and evidence-informed visibility for the link worker model; by presenting articles in respected journals and newspapers, the initiative gains recognition within professional communities and aligns with broader healthcare or social care priorities | In the short term, this exposure increases awareness and stimulates professional curiosity | In the medium term, it generates higher demand for knowledge, training, and intersectoral collaboration related to the link worker role | Over time, this sustained professional engagement leads to formal integration of the link worker approach into organizational and clinical practice, strengthening adoption, penetration, and sustainability of the model within relevant systems |
| Medical journals: Develop and distribute educational materials; peer-reviewed dissemination to GPs | This strategy focuses on the development and dissemination of peer-reviewed educational materials in relevant medical journals aimed at GPs and other healthcare professionals; the activation of this strategy requires the production of evidence-based content that clearly articulates the purpose, mechanisms, and outcomes of the link worker model, as well as access to suitable publication channels within the medical community | The effectiveness of this approach is moderated by the credibility of the journal, the perceived scientific rigour of the publication, and the relevance of the topic to clinical practice | The mechanism operates through the formal dissemination of validated knowledge, which builds professional legitimacy and enhances clinicians’ confidence in referring patients to or collaborating with link workers; by embedding the intervention in peer-reviewed literature, the strategy supports knowledge translation and alignment with established medical standards | In the short term, this results in increased awareness and understanding of the link worker model among GPs and primary care professionals | In the medium term, it promotes evidence-informed decision-making, improved referral practices, and greater integration of the link worker role within clinical pathways | Over time, these developments strengthen the adoption, fidelity, and sustainability of the link worker model within healthcare systems, as the approach becomes embedded in routine practice and professional education |
| Posts on LinkedIn: Use mass media (social media is explicitly included here) | This strategy presupposes an active and well-maintained LinkedIn profile, along with a relevant professional network that includes health, social care, and community stakeholders | Its effectiveness is moderated by factors such as the frequency and quality of posts, algorithmic exposure, and levels of engagement (e.g. likes, shares, and comments) | The mechanism functions through enhanced digital visibility and the creation of professional dialogue around the link worker model; by sharing updates, success stories, and evidence-based insights, LinkedIn posts facilitate the exchange of knowledge and strengthen professional legitimacy across sectors | In the short term, this generates increased interaction and interest from professionals, organizations, and policy-makers | In the medium term, it promotes cross-sector collaboration and knowledge sharing, helping to build a shared understanding of the link worker’s role and impact | Over time, these network-based interactions enhance professional recognition and normalize the inclusion of the link worker approach within wider service systems: this process supports acceptability, adoption, and penetration of the model by embedding it within professional discourse and everyday practice |
| Podcasts: Use mass media | This strategy involves the production and dissemination of podcast episodes to communicate the purpose and value of the link worker model to both professionals and the wider public; activation requires access to suitable production resources and podcast platforms, as well as relevant guests, such as link workers, service users, or partner organizations, who can share insights and experiences | The effectiveness is moderated by the quality of the podcast content, frequency, and ability to reach target audiences | The mechanism operates through storytelling and accessible communication, which humanizes the link worker role and promotes engagement across sectors |  |  |  |
| TV2 Kosmopol (a regional TV segment): Use mass media | A key precondition for activating mechanisms related to the TV2 Kosmopol segment was the general lack of awareness and understanding of social prescribing (SP) among professionals and the wider public | By using a trusted regional media platform, the project sought to introduce the concept in a credible and relatable way | The effectiveness of this mechanism was moderated by how the story was framed, its local relevance, authenticity of patient or professional voices, and the credibility of the spokespersons; the mechanism activated through this exposure was public recognition and legitimisation: when SP was presented as a meaningful, community-based health innovation, audiences began to perceive it as real, necessary, and aligned with existing health priorities | The short-term outcome was increased awareness and curiosity about SP among healthcare actors and citizens | This, in turn, contributed to medium-term outcomes such as strengthened stakeholder engagement and openness to collaboration across sectors | The long-term implementation outcome linked to this strategy was adoption and acceptability: the sense that SP belongs within local health and social care systems |
| Webinar on SP: Conduct educational meetings | The webinar strategy built on the precondition that potential implementers and partners were interested in SP but lacked a concrete understanding of its operational elements | The online educational meeting created a structured opportunity for shared learning, moderated by the quality of facilitation, interactivity, and the relevance of the cases discussed | The central mechanism was collective learning and meaning-making: through exposure to examples and dialogue with peers, participants could envision how SP might work in their own setting; this enhanced perceived relevance and self-efficacy, key psychological drivers of change | The short-term outcome was increased knowledge and conceptual clarity regarding SP processes and benefits | As participants reflected on local applicability, motivation and readiness for change increased, which led to medium-term outcomes such as the initiation of small-scale trials or planning activities within their organizations | The long-term implementation outcomes supported by this strategy were feasibility and appropriateness: an increasing belief that SP could be realistically and meaningfully integrated into practice |
| Website about the SP project: Develop and distribute educational materials | For sustained implementation, a key precondition was the need for easily accessible, continuously updated information about SP; the project website addressed this by offering a central hub for educational materials, evidence summaries, and project updates | The success of this mechanism depended on moderators such as the usability of the platform, clarity of language, and frequency of updates | The main mechanism at play was ongoing sense-making and reinforcement: by revisiting trustworthy information and resources, users could consolidate understanding and sustain their engagement beyond initial exposure | The short-term outcome was improved comprehension of SP’s conceptual underpinnings and methods | Over time, this supported medium-term outcomes such as strengthened inter-organizational alignment and coherence in practice | In the longer term, the website contributes to the sustainability and fidelity of implementation, helping ensure that SP principles are maintained and refined across sites as the initiative matures |
| Policy strategies | | | | | | |
| Presentation to political committees: Socialist People’s Party’s Health Committee: Build a coalition (mobilize political actors) + Mandate change (if the goal is new policies) | A core precondition for this strategy was the need to establish political legitimacy and ownership for SP within parties that prioritize public health and equity; the presentation aimed to mobilize political actors who could champion SP as a policy innovation | The process was moderated by the committee’s current policy agenda, the framing of SP in relation to party values, and the clarity of the evidence base presented | The mechanism activated was coalition-building through shared framing; positioning SP as aligned with social justice, prevention, and local empowerment created a sense of political resonance | In the short-term, this generated political interest and support statements | Medium-term outcomes included integration of SP into party health policy discussions and draft proposals | The long-term implementation outcomes were mandated change and political commitment, laying the groundwork for policy endorsement and future resource allocation |
| Presentations to the Moderates’ Health Secretariat: Inform local opinion leaders (political staff as influencers) + Build a coalition | This strategy responded to the precondition that cross-party awareness was essential for broad policy traction | The Moderates’ Secretariat was approached as a key opinion-shaping body within the political system | The mechanism depended on framing SP as an evidence-based, pragmatic initiative consistent with centrist health policy goals (efficiency, prevention, patient involvement); by informing and engaging political staff as influencers, the mechanism of elite cueing was activated, where informed advisors shape party narratives | Short-term outcomes included enhanced understanding and positive framing of SP among policy staff | Medium-term outcomes were references to SP in internal discussions and policy briefs | The long-term outcome was coalition expansion with a widening base of cross-party support, contributing to the future adoption of SP in policy agendas |
| Presentations to the Capital Region’s Prevention Department: Change service context (regional policy alignment) + Build a coalition | The precondition here was the need for regional alignment and system-level integration to sustain SP across localities | Moderators included the department’s strategic priorities, existing prevention frameworks, and openness to partnership | The mechanism was policy alignment through dialogue; by connecting SP with the region’s prevention and health promotion goals, the presentation encouraged shared ownership and perceived fit within existing policy structures | Short-term outcomes included recognition of SP’s relevance to regional priorities | medium-term outcomes were exploration of collaborative projects or resource-sharing | The long-term implementation outcome was a change in service context, an enabling policy environment supporting SP’s institutionalization |
| Participation at People’s Political Festival 2025: Use mass media (broad dissemination to public/political audience) + Build a coalition (networking with policy stakeholders) | The festival offered a visible, public platform, a precondition for broad dissemination and legitimacy across sectors | Moderators included media interest, the composition of the political audience, and opportunities for informal dialogue | Mechanisms included public visibility (through media exposure) and networked coalition-building (through direct engagement with stakeholders and citizens) | Short-term outcomes were increased awareness of SP among politicians, non-government organizations, and the public | Medium-term outcomes included new collaborations, invitations to policy dialogues, and strengthened political networks | The long-term outcomes were growth of the coalition and public acceptability, both crucial for long-term adoption of SP as part of the mainstream prevention policy |
| Networking with potential donors: Access new funding (ERIC’s financial strategy cluster), often paired with Build a coalition | The precondition was recognition that sustainable implementation requires financial commitment beyond pilot funding | Moderators included donor priorities, clarity of the value proposition, and credibility of the project leadership | The mechanism activated was coalition-building through financial alignment; engaging funders as strategic partners who see SP as advancing their mission (e.g. social cohesion, health equity) | Short-term outcomes were expressions of interest or exploratory meetings | Medium-term outcomes involved funding applications or letters of intent | The long-term implementation outcome was access to new funding, enhancing the sustainability and scalability of SP |
| Presentation at the Loneliness Conference at the Danish Parliament: Build a coalition + Inform local opinion leaders | A key precondition was that loneliness had become a prominent political concern, offering a receptive entry point for discussing SP; the presentation targeted policy stakeholders and opinion leaders already engaged in social and health issues | Moderators included the conference’s political tone, speaker lineup, and opportunities for media coverage | The mechanism was knowledge mobilization in a policy forum; by linking SP to an urgent social problem, the initiative gained emotional and political salience | Short-term outcomes were increased policy awareness of SP as a relevant response to loneliness | Medium-term outcomes included potential partnerships with ministries, non-government organizations, or municipalities | The long-term implementation outcomes were coalition strengthening, policy endorsement, and steps towards system-level adoption |
| Develop stakeholder inter-relationships | | | | | | |
| Peer group meeting: Create a learning collaborative | Professionals across practices experience similar challenges in translating SP into daily routines but lack a structured forum for shared reflection | Level of trust between participants, quality of facilitation, stability and continuity of meetings | Collective reflection and mutual learning, practitioners share experiences, co-construct knowledge, and normalize new practices through peer dialogue | Enhanced peer support, shared vocabulary, and improved confidence in applying SP principles | Coordinated approaches and shared ownership of implementation across participating practices | Fidelity and sustainability, consistency, and enduring use of SP practices across sites |
| Expert visit by the link worker in general practices: Build a coalition | Limited understanding of the link worker’s role and uncertain collaboration pathways between primary care and community services | Openness of practice staff, time availability, perceived legitimacy and competence of the link worker | Relational trust-building and clarification of roles; personal interaction breaks down misconceptions and builds a sense of partnership | Improved understanding of the link worker’s function and increased willingness to collaborate | Integration of referral pathways and cross-sector coordination within the practice workflow | Adoption and appropriateness: SP viewed as a relevant, embedded part of primary care |
| Medical experts as ambassadors for the intervention: Identify and prepare champions | Clinicians often rely on respected peers to validate new approaches before changing their own practice | Credibility and professional status of ambassadors, their communication skills, and alignment with institutional values | Professional modelling and normative influence by trusted experts publicly endorse SP, reducing perceived risk and increasing legitimacy | Raised awareness and positive professional attitudes towards SP | Diffusion of SP practices through informal peer networks and mentoring | Adoption and acceptability: SP gains professional legitimacy and becomes standard within clinical culture |
| Interest in group collaboration through cluster meetings: Create a learning collaborative (it is about systematically bringing several practices together in clusters to exchange experiences, support each other, and co-create solutions) | Individual practices are motivated but isolated; they need structured interaction to maintain momentum and problem-solve collectively | Group diversity, facilitation style, balance between structure and openness, continuity of participation | Collective problem-solving and co-creation; practices exchange strategies, benchmark progress, and develop shared local solutions | Improved insight into common challenges and effective adaptations | Cross-practice coordination and joint refinement of SP delivery models | Penetration and coherent uptake of SP across practices within the region |
| The link worker at the library: Promote networking | Implementation of SP depends on linking health services with community resources in accessible, non-clinical spaces | Visibility of the link worker, accessibility of the site, local stakeholder engagement, and community awareness | Networking and boundary spanning, where the link worker connects diverse actors, fosters informal collaborations, and builds social capital across sectors | New partnerships and increased referral opportunities between health, social, and community actors | Strengthened cross-sector collaboration and integration of SP within local service networks | Reach and sustainability: SP embedded in the wider community ecosystem beyond clinical settings |
| Presentations at various professional associations, e.g. annual meeting of the Danish College of General Practitioners: Build a coalition (a strategy aimed at bringing together a broader group of professional stakeholders to achieve legitimacy, support, and a shared direction, without necessarily focusing on education or capacity-building) | Implementation of SP requires legitimacy and shared understanding among professional stakeholders such as GPs and other primary care providers; however, awareness of SP and its evidence base is still limited within professional associations | Relevance of SP to the professional audience’s priorities (e.g. workload, patient care, prevention), perceived credibility of presenters, framing of the intervention as aligned with professional values, and opportunities for interaction or endorsement | Coalition-building through professional alignment; by presenting SP within established professional forums, the initiative gains legitimacy, fosters identification with existing professional missions, and positions SP as a credible and evidence-informed innovation rather than an externally imposed agenda | Increased awareness, recognition, and initial buy-in among professional members; SP becomes part of professional discourse and curiosity | Emergence of supportive professional networks and informal advocacy for SP within associations; identification of potential champions who can integrate SP perspectives into guidelines, training, or continuing education | Adoption and acceptability: SP becomes recognized as a legitimate and professionally endorsed approach within the broader primary care and public health community, paving the way for system-level integration |
| The link workers host a networking meeting with local and municipal services: Build a coalition (the networking meeting is not merely an information event but a strategic effort to build a coalition, where SP becomes recognized, supported, and integrated into local structures) | Successful implementation of SP depends on cross-sector collaboration, yet local and municipal services often operate in parallel systems with limited coordination or shared understanding of roles; there is a need to establish mutual recognition and trust across these boundaries | Level of existing collaboration between services, representation of relevant actors, leadership engagement, clarity of meeting purpose, and quality of facilitation; local political context and organizational readiness can further shape outcomes | Coalition-building through structured relational engagement; by convening actors from health, social, and community sectors, the link workers facilitate mutual understanding, highlight shared goals, and create a sense of collective ownership of SP as a local initiative rather than an external project | Increased mutual awareness of roles and resources; strengthened relationships and communication channels between local and municipal stakeholders | Development of collaborative agreements or shared referral pathways; increasing institutional support and alignment of SP within local governance and service structures | Adoption, appropriateness, and sustainability: SP becomes formally recognized, supported, and embedded within local cross-sector systems, ensuring long-term viability and integration |
| Train and educate stakeholders | | | | | | |
| Link worker visits/tests activists – recommendations: Conduct educational outreach visits (the purpose is knowledge/skill-building) | GPs and community actors show interest in SP but lack hands-on knowledge and confidence to refer or operationalize the model | Availability of staff, perceived relevance of SP, communication skills of the link worker, and level of pre-existing relationships | Experiential learning through outreach by visiting and engaging directly with practitioners; the link worker translates SP principles into practical steps, tailors guidance, and reinforces learning through real cases | Improved understanding of SP workflows and increased confidence in referral decision-making | More consistent and accurate use of SP referrals; better integration of the link worker into practice routines | Fidelity and feasibility: SP practices are applied correctly and perceived as manageable within daily workflows |
| Feedback loop: awareness and reminders: Remind clinicians | Even after initial engagement, busy clinicians may forget or deprioritize SP referrals amid competing clinical demands | Frequency, timing, and format of reminders (e.g. e-mail, prompts in electronic health records, meeting updates); perceived helpfulness and tone of communication | Cue to action and cognitive reinforcement by timely reminders sustain awareness and normalize SP as part of standard care routines | Renewed attention to SP and maintenance of referral activity | Stabilization of SP practice patterns and prevention of implementation decay | Fidelity and sustainability: SP remains part of routine practice over time |
| Focus on evaluation, reflection, and learning loops in the project plan: Audit and provide feedback | Teams need feedback on their progress to recognize successes, identify barriers, and sustain motivation | Credibility and clarity of data, timing and tone of feedback, and openness to reflective discussion | Learning through reflection on the data; systematic feedback on referrals, outcomes, and processes fosters adaptive learning and accountability | Increased insight into performance and recognition of areas for improvement | Iterative refinement of SP workflows and strengthened learning culture within teams | Fidelity, appropriateness, and sustainability: continuous improvement ensures long-term quality and alignment with local needs |
| Meeting with clinic staff: Conduct educational meetings | Clinic staff need a shared understanding of the SP concept, roles, and referral procedures to coordinate effectively | Facilitation style, inclusivity of discussion, perceived relevance to daily practice, and staff turnover | Interactive learning and sense-making; educational meetings provide a space for clarifying questions, aligning expectations, and building shared ownership | Improved team understanding and motivation to engage in SP | Strengthened internal communication and smoother coordination of referrals | Adoption and fidelity: SP becomes embedded as a coordinated team activity |
| Stratifying contact with GPs (based on referral activity): Facilitation or Use data experts; Develop stakeholder inter-relationships (because it tailors engagement) | GPs vary in their engagement and referral activity; the same communication to all may be inefficient | Access to referral data, capacity for targeted communication, and openness of GPs to tailored support | Targeted facilitation based on data insight using referral patterns to identify low- and high-engagement practices allows tailored dialogue, problem-solving, and recognition | More efficient allocation of support and increased responsiveness from less active practices | Balanced and equitable participation across GP practices | Reach and fidelity: improved coverage and consistency of SP implementation across the network |
| Introductory meet-and-greet rounds with materials and lunch meetings at GP clinics: Conduct educational outreach visits or Conduct educational meetings | Initial awareness of SP among clinic staff is low, and early relational trust needs to be established before more complex collaboration can occur | Informal atmosphere, accessibility of materials, openness of staff, and presence of key champions | Relationship-based learning and engagement; informal introductions build rapport, convey essential information, and create psychological safety for future collaboration | Increased familiarity with the link worker and initial understanding of the purpose of SP | Emerging readiness to participate in referrals and discussions about integration | Acceptability and adoption: SP becomes recognized as a legitimate and welcomed component of clinical care |
| Change infrastructure | | | | | | |
| GP develops referral/inclusion guidelines adapted to 5 different electronic systems (phrases): Change record systems; Develop and implement tools for quality improvement | Variation across electronic record systems makes it difficult for GPs to document and refer patients consistently to SP | Compatibility of the electronic systems, GP engagement in co-design, usability of guidelines, and IT support availability | Standardization through technical adaptation, creating adaptable referral phrases and templates across systems ensures that all GPs can operationalize SP within their existing digital workflows | Increased ease of referral and consistency in patient inclusion criteria | Improved data capture, cross-practice comparability, and reduced administrative barriers | Fidelity and feasibility: SP integrated smoothly within the existing digital infrastructure, supporting sustainable use |
| Referral/triage module for GPs: Change record systems; Alter patient/clinician information systems | Referrals to SP are often informal and untracked, limiting accountability and coordination between GPs and link workers | User interface design, interoperability with existing systems, and training provided to clinicians | Workflow integration through system modification, embedding a referral/triage module automates processes, provides feedback loops, and normalizes SP as part of routine electronic workflows | Simplified referral process and improved communication between GPs and link workers | More systematic data exchange and monitoring of referral patterns | Fidelity and sustainability: reliable referral processes institutionalized within the information system |
| Need for a consolidated list of referral options; overview of associations: Develop resource-sharing agreements; Develop and implement tools | GPs and link workers lack a clear, up-to-date overview of available community services and referral options | Frequency of updates, clarity of layout, accessibility (digital/print), and stakeholder input in maintaining the list | Resource visibility and network mapping; creating a shared overview increases confidence, reduces uncertainty, and enables timely referrals | Improved navigation of community resources; fewer missed or inappropriate referrals | Enhanced coordination between primary care and community organizations | Reach and appropriateness: SP referrals align more effectively with patient needs and available services |
| Develop resource-sharing agreements and develop and implement tools: Change record systems + overlap with Remind clinicians | Siloed data systems and unclear information-sharing practices hinder continuity between clinical and community partners | Legal and organizational policies, data privacy regulations, technical interoperability, and trust between partners | Formalization of shared processes; resource-sharing agreements and interoperable tools establish accountability, standard communication, and reminders that sustain collaboration | Clearer referral pathways and improved data flow | Regular, transparent exchange of information between sectors | Sustainability and fidelity: inter-organizational structures support long-term, consistent delivery of SP |
| Intervention manual (guidebook) for link workers’ visits to services: Develop and implement tools for quality improvement | Link workers vary in their approach to outreach and collaboration with community services, leading to inconsistent quality | Clarity of the manual, relevance to the practice context, link worker training, and adaptability to local variation | Codification of practice knowledge, providing a structured guide supports standardization and maintains flexibility, ensuring consistent quality across link worker activities | Improved confidence and coherence in how link workers conduct visits | Greater alignment in partnership-building and outreach methods | Fidelity and quality improvement: standardized yet adaptable practice across link worker activities |
| Description/outline of the link worker function: Revise professional roles | The link worker role is new and unfamiliar to many health and community professionals, creating uncertainty about responsibilities and scope | Clarity and communication of role description, managerial support, and integration into existing team structures | Professional role clarification; defining tasks, competencies, and boundaries legitimizes the link worker as a distinct but collaborative role | Shared understanding of the link worker’s purpose and contribution | Improved interdisciplinary coordination and smoother referral processes | Acceptability and adoption: link worker function becomes institutionalized within the service system |
| Description/outline of the link worker pathway (including workflows, documentation phrases, networks, methods for specific groups, transition phases, etc.): Develop and implement tools; Revise professional roles; Change record systems | The link worker’s tasks and documentation methods vary across sites, reducing coherence and data comparability | Involvement of practitioners in co-design, compatibility with digital systems, and support for training and adaptation | Process standardization and role consolidation; describing workflows, documentation templates, and network activities ensures alignment across SP delivery | Clearer processes for referral, follow-up, and transitions between services | More coordinated practice, improved monitoring, and higher data quality | Fidelity, appropriateness, and sustainability: consistent SP pathways embedded in local and digital systems |
| Develop status overview and registration sheets for the link worker pathway: Develop and organize quality monitoring systems | Ongoing quality monitoring is necessary, but a standardized method for tracking link worker activities and outcomes is lacking | Ease of use, clarity of indicators, alignment with reporting requirements, and integration into daily workflow | Feedback through structured monitoring, registration sheets make progress visible, enable reflection, and support data-informed adjustments | Improved documentation and awareness of performance | Use of monitoring data to guide quality improvement and strategic learning | Fidelity, quality, and sustainability: systematic monitoring supports continuous improvement and accountability |
| Use evaluative and iterative strategies | | | | | | |
| Feedback loop with patient engagement (feedback from patients): Audit and provide feedback | Ongoing patient engagement is central to SP, but staff often lack systematic insight into patient experiences and the perceived value of the intervention | Frequency and quality of patient feedback collection, methods used (surveys, interviews, informal feedback), and openness of staff to reflection | Learning through user feedback; patient perspectives provide concrete, emotionally resonant insights that enhance staff motivation, empathy, and sense of purpose | Increased understanding of patient experiences and perceived impact of SP | Adaptations in practice based on feedback (e.g. more personalized support, improved communication) | Appropriateness and acceptability: SP becomes more responsive to patient needs and is perceived as valuable by both users and providers |
| Feedback loop with workload management: Audit and provide feedback | Implementation requires balancing SP activities with existing clinical workloads; without insight into workload effects, engagement can decline | Accuracy of workload data, transparency in feedback discussions, and managerial commitment to address workload concerns | Reflective feedback for workload calibration; systematic review of workload data helps identify bottlenecks and redistribute responsibilities more fairly | Recognition of workload challenges and evidence-based adjustments to task allocation | Improved staff satisfaction, reduced stress, and enhanced perception of SP as manageable | Feasibility and sustainability: SP integrated into daily practice without overburdening staff |
| Feedback loop with awareness and reminders: Remind GPs | After initial implementation, attention to SP can fade without prompts that reinforce its presence in everyday routines | Mode and timing of reminders (e-mail, electronic health record notifications, meeting notes), perceived relevance, and tone of communication | Cognitive cueing and reinforcement; reminders trigger recall, maintain visibility, and signal ongoing institutional support for SP | Renewed attention and continued referral activity among GPs | Maintenance of stable SP participation rates and avoidance of implementation drift | Fidelity and sustainability: SP remains part of standard clinical behaviour over time |
| Integration of reminders into annual review forms for GPs/nurses: Remind clinicians + Change record systems (because reminders are built into forms/electronic systems) | Manual reminders risk being overlooked; embedding SP prompts into electronic or standardized annual review forms ensures systematic consideration | Quality of integration within IT systems, perceived usefulness of the prompt, and clinician attitudes toward digital reminders | Automation of cueing and normalization through system design; SP reminders built into forms institutionalize the behaviour by linking it to existing quality review routines | Increased recall and routine inquiry about SP during annual patient reviews | Normalization of SP as part of preventive care documentation | Fidelity and sustainability: SP embedded in the formal record system and aligned with professional review processes |

# Supplementary Table 3. Distribution of the implementation strategies across the actor groups

| ERIC cluster | Strategy | General practitioners | Link workers | Volunteer Centre | DaneAge Association |
| --- | --- | --- | --- | --- | --- |
| Dissemination | Newspaper articles: Use mass media | x |  |  |  |
|  | Website: Use mass media; Public visibility | x | x | x |  |
|  | Develop new materials for the extended target group 18+ years, including a new link worker: Develop and distribute educational materials |  | x | x |  |
|  | Request for a video introducing the link worker (not yet developed): Develop and distribute educational materials (video as an educational tool) |  |  |  |  |
|  | E-mails from an implementation champion (a GP): Conduct educational outreach visits | x |  |  |  |
|  | Articles in Occupational Therapy Magazine or other professional journals and newspapers: Use mass media) | x |  |  |  |
|  | Medical journals: Develop and distribute educational materials (peer-reviewed dissemination to GPs) | x |  |  |  |
|  | Posts on LinkedIn: Use mass media (social media is explicitly included here) | x |  |  | x |
|  | Podcasts: Use mass media | x |  |  |  |
|  | Regional TV segments: Use mass media |  | x | x | x |
|  | Webinar on social prescribing: Conduct educational meetings | x | x | x |  |
|  | Website about the social prescribing project: Develop and distribute educational materials | x | x | x | x |
| Policy strategies | Presentation to political committees: Build a coalition (mobilize political actors) + Mandate change (if the goal is new policies) | x |  |  | x |
|  | Presentations to the Moderates’ Health Secretariat: Inform local opinion leaders (political staff as influencers) + Build a coalition | x |  |  |  |
|  | Presentations to the Capital Region’s Prevention Department: Change service context (regional policy alignment) + Build a coalition | x |  |  | x |
|  | Participation at People’s Political Festival 2025: Use mass media (broad dissemination to public/political audience) + Build a coalition (networking with policy stakeholders) | x |  |  | x |
|  | Networking with potential donors: Access new funding (ERIC’s financial strategy cluster), often paired with Build a coalition | x |  | x | x |
|  | Presentation at the Loneliness Conference at the Danish Parliament: Build a coalition + inform local opinion leaders | x | x | x | x |
| Develop stakeholder inter-relationships | Peer group meeting: Create a learning collaborative |  | x | x |  |
|  | Expert visit by the link worker in general practices: Build a coalition |  | x |  |  |
|  | Medical experts as ambassadors for the intervention: Identify and prepare champions | x |  |  |  |
|  | Interest in group collaboration through cluster meetings: Create a learning collaborative (it is about systematically bringing several practices together in clusters to exchange experiences, support each other, and co-create solutions) | x |  |  |  |
|  | The link worker at the library: Promote networking |  | x |  |  |
|  | Presentations at various professional associations, e.g. annual meeting of the Danish College of General Practitioners: Build a coalition (a strategy aimed at bringing together a broader group of professional stakeholders to achieve legitimacy, support, and a shared direction, without necessarily focusing on education or capacity-building) | x |  |  |  |
|  | The link workers host a networking meeting with local and municipal services: Build a coalition (the networking meeting is not merely an information event but a strategic effort to build a coalition, where social prescribing becomes recognized, supported, and integrated into local structures) |  | x | x | x |
| Train and educate stakeholders | Link worker visits/tests activists – recommendations: Conduct educational outreach visits (the purpose is knowledge/skill-building) |  | x | x | x |
|  | Feedback loop to generate awareness with reminders: Remind clinicians | x |  |  |  |
|  | Focus on evaluation, reflection, and learning loops in the project plan: Audit and provide feedback | x | x | x | x |
|  | Meeting with clinic staff: Conduct educational meetings |  | x |  |  |
|  | Stratifying contact with GPs (based on referral activity): Facilitation or use data experts; Develop stakeholder inter-relationships to tailor engagement |  | x |  |  |
|  | Introductory meet-and-greet rounds with materials and lunch meetings at GP clinics: Conduct educational outreach visits or Conduct educational meetings |  | x | x |  |
| Change infrastructure | GP develops referral/inclusion guidelines adapted to 5 different electronic systems (phrases): Change record systems; Develop and implement tools for quality improvement | x |  |  |  |
|  | Referral/triage module for GPs: Change record systems; Alter patient/clinician information systems | x |  |  |  |
|  | Need for a consolidated list of referral options/overview of associations: Develop resource-sharing agreements; Develop and implement tools |  | x | x | x |
|  | Develop resource-sharing agreements: Develop and implement tools; Change record systems + overlap with Remind clinicians |  |  | x | x |
|  | Intervention manual (guidebook) for link workers’ visits to services: Develop and implement tools for quality improvement |  | x | x | x |
|  | Description/outline of the link worker function: Revise professional roles |  | x | x | x |
|  | Description/outline of the link worker pathway (including workflows, documentation phrases, networks, methods for specific groups, transition phases, etc.): Develop and implement tools; Revise professional roles; Change record systems |  | x | x | x |
|  | Develop status overview and registration sheets for the link worker pathway: Develop and organize quality monitoring systems |  | x | x |  |
| Use evaluative and iterative strategies | Feedback loop involving patient engagement (feedback from patients): Audit and provide feedback |  | x |  |  |
|  | Feedback loop on workload management: Audit and provide feedback |  | x | x |  |
|  | Feedback loop on awareness and reminders: Remind GPs | x |  |  |  |
|  | Integration of reminders into annual review forms for GPs/nurses: Remind clinicians + Change record systems (because reminders are built into forms/electronic systems) |  | x | x |  |

GP, general practitioner.
